# Evidence of transitioning apple farming to an agro-ecological model in Himachal Pradesh

**DOI:** 10.3389/fnut.2025.1611137

**Published:** 2025-06-24

**Authors:** Divyanshu, Subhash Sharma, Rajeshwar Singh Chandel, Rohit Vashishat, Subhash Chander Verma, Sudhir Verma, Narender Kumar Bharat, Kuldeep Singh Thakur, Inder Dev, Sanjeev Chauhan, Ashu Chandel, Kamal Kishore, Ajay Kumar

**Affiliations:** Dr. Yashwant Singh Parmar University of Horticulture and Forestry, Nauni, India

**Keywords:** natural farming, soil health, pest management, agroecology, profitability

## Abstract

Agro-ecological farming practices that integrate economic viability and environmental resilience, based on principles designed to support optimal food and nutritional security in farming systems. This study compares Natural Farming (NF) and Conventional Farming (CF) in apple orchards of Himachal Pradesh by using agro-ecological indicators to assess soil health, pest management, and profitability. The research was conducted across the High Hills Temperate Wet (Zone III) and Dry Temperate High Hills (Zone IV). A total of 140 farm (70 under Natural Farming and 70 under Conventional Farming) were sampled by using simple random sampling method and Structural Equation Modeling (SEM) was used to analyze the interrelationships among soil nutrient status, pest and disease incidence, and farm profitability. The results revealed that NF had higher organic carbon (OC) levels, ranging from 0.84 to 1.95%, compared to CF, which ranged from 0.53 to 1.91%. NF also exhibited higher nitrogen (N), phosphorus (P), and micronutrient levels, while potassium (K) levels were lower. Pest incidence was significantly higher in NF, with Woolly Apple Aphid (50.08%) and Leaf Folder (41%) infestations, compared to CF (17.5 and 5.5%, respectively). NF also showed a 1.59% increase in yield (161.25 quintals/ha) and a 46.76% reduction in total variable costs. Structural equation modeling (SEM) identified key pathways linking farming practices to soil quality, yield performance, and economic outcomes. The analysis revealed that organic matter positively influenced microbial activity (0.05), thereby enhancing soil fertility. SEM findings also highlighted the importance of balanced nutrient management for sustaining both productivity and profitability. These results underscore NF’s capacity to support agro-ecological indicators by enhancing both economic and environmental resilience, while encouraging long-term nutritional security through this agro-ecological supported system. This research provides compelling evidence for adopting NF as a transformative approach in apple farming systems.

## 1 Introduction

Agroecology offers a holistic way of farming that blends ecological principles with agricultural practices to promote sustainability, protect biodiversity, and improve soil health: all while reducing the need for chemical inputs (1, 2). As environmental challenges and food security concerns increase globally, there is growing interest in farming systems that not only maintain productivity but also build ecological resilience (3–7). Earlier, Zero Budget Natural Farming (ZBNF) was widely recognized in India, but it has since been renamed Natural Farming (NF) to reflect the understanding that farming, by nature, involves some costs. NF is a broader agroecological system that incorporates low external inputs, intercropping, and biological controls. It has shown promising results in cereal and vegetable production, with farmers reporting improved crop health, better soil quality, and more sustainable outcomes. However, applying NF principles to perennial crops like apple has proven more complex. Unlike short-duration crops, apple orchards require long-term care and are more susceptible to diseases over time, raising the important question of whether NF can be as effective in apple cultivation as it is in other crops.

In Himachal Pradesh, NF gained formal support with the launch of the Prakritik Kheti Khushal Kisan Yojna (PK3Y) in 2018. Since then, many farmers have adopted NF methods, not only for seasonal crops but also for apples, a key pillar of the state’s economy. This shift highlighted the need for more research on how NF impacts soil health, disease incidence, and farm profitability. However, conventional practices, which often depend heavily on chemical fertilizers and pesticides, have raised concerns about soil degradation, input dependency, and the sustainability of the system in the long run (8–11). This has led to a declining trend in crop yield growth, often linked to the overuse of synthetic fertilizers and pesticides (12, 13). While some studies suggest a reduction in yields due to chemical-intensive practices (14–16) others report no significant decline (17, 18). Simultaneously, increasing health consciousness among consumers has driven demand for chemical-free produce (19).

A major strength of NF is its positive impact on soil health, as it promotes heterotrophic microbial growth and boosts soil organic matter, both vital for long-term productivity (14, 20–23). An important distinction between NF and CF lies in disease management, where CF relies on frequent synthetic pesticide use to achieve short-term control and lower immediate disease incidence (24). However, this chemical dependency often leads to the development of resistance among pathogens and disrupts the balance of beneficial soil and plant-associated microbes (25). In contrast, NF avoids synthetic chemicals and instead emphasizes ecological balance and biological control. It promotes the use of indigenous bio-pesticide formulations such as *Neemaster*, *Agniaster*, and *Bramhaster*, which are derived from natural sources and are environmentally benign (26, 27). This integrated system not only supports healthier crops but also contributes to a more resilient and self-sustaining agroecosystem.

Beyond environmental concerns, economic sustainability remains a major challenge for apple farmers. Agrarian distress, driven by low price realization, rising input costs, and increasing climatic uncertainties, has steadily eroded farm incomes (28). In this context, NF offers a potential pathway toward economic resilience by lowering input dependence and promoting long-term productivity. However, despite its growing adoption, there is a conspicuous lack of systematic, empirical evidence comparing the agroecological and economic outcomes of NF and CF in apple cultivation, particularly within the context of Himachal Pradesh’s varied agro-climatic zones. Most existing studies have focused narrowly on CF’s impact or have examined NF in short-duration crops, leaving a critical void in understanding its effectiveness in perennial, high-value crops like apple. Furthermore, findings on NF’s influence on yield and profitability remain inconsistent across regions and crop types, offering little actionable insight for policymakers or farmers. This study addresses this gap by comprehensively evaluating the comparative effects of NF and CF on soil nutrient status, disease incidence and severity, and farm profitability in apple orchards, a dimension of research largely unexplored in the Indian Himalayan context. The study aimed to: (i) compare the impact of NF and CF on soil properties and nutrient content in apple orchards, (ii) evaluate disease incidence and severity under both systems, and (iii) assess the profitability of NF versus CF apple orchards.

## 2 Methodology

### 2.1 Study area and sampling technique

**Figure 1:**
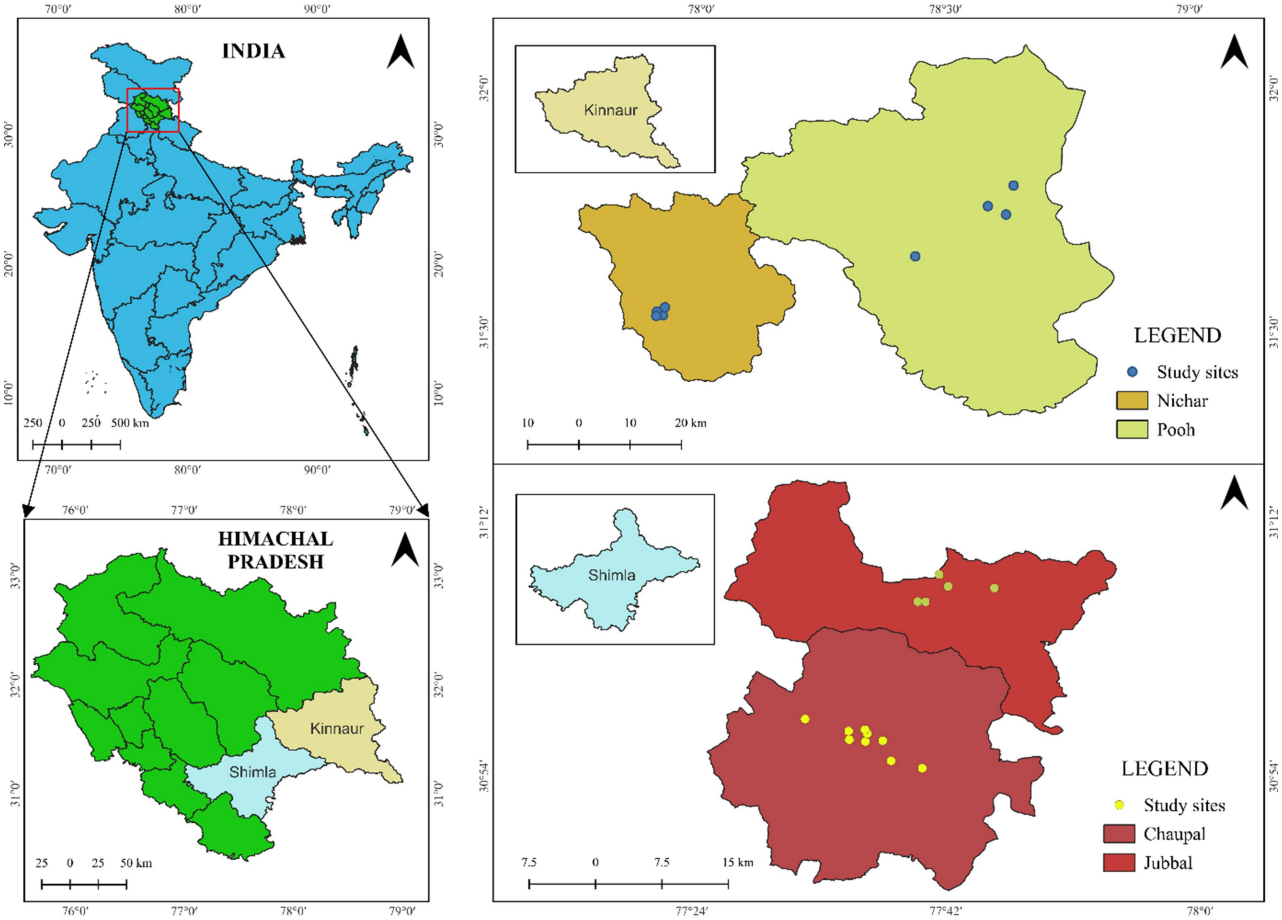
Location map of the study area.

The study was conducted in Himachal Pradesh, India, situated in the North-Western Himalayas. This region extends between latitudes 30° 22′40′′ N and 33° 12′20′′ N and longitudes 75° 45′55′′ E and 79° 04′20′′ E, with elevations ranging from 350 to 6,975 meters above mean sea level, resulting in diverse climatic conditions. A simple random sampling approach was employed to select the farmers for the study. Himachal Pradesh is classified into four agro-climatic zones based on elevation, ranging from below 650 meters to over 2,200 meters above mean sea level. The study focused on two key apple-producing zones: the High Hills Temperate Wet Zone (Zone III) and the Dry Temperate High Hills Zone (Zone IV). To ensure a representative sample, a comprehensive list of blocks within these zones was compiled. Two blocks with the highest apple production were selected from each zone, resulting in the choice of Chaupal and Jubbal blocks in Shimla district and Nichar and Pooh blocks in Kinnaur district (Figure 1). From each block, 35 farmers’ fields were randomly selected, bringing the total sample size to 140 apple farms. Of these, 70 farms were under Natural Farming (NF), while the remaining 70 followed Conventional Farming (CF).

### 2.2 Collection of soil samples

Soil samples were collected systematically from each farm using the composite soil sampling method, which involved gathering multiple subsamples from different locations within the field to account for soil variability. To ensure a representative sample, five subsamples were randomly taken from a depth of 0 to 20 cm in each farmer’s field following a zigzag sampling pattern. These subsamples were then mixed thoroughly to form a single composite sample for analysis. The study aimed to assess the impact of NF and CF on various soil properties, including pH, electrical conductivity (EC), soil organic carbon (OC), and the availability of macronutrients (N, P, K) and micronutrients (Fe, Mn, Zn, Cu). After collection, the composite samples were shade-dried, gently crushed using a wooden pestle and mortar, and passed through a 2 mm sieve. The processed soil samples were then stored in polyethylene bags for further laboratory analysis (29, 30).

### 2.3 Nutrient and enzymatic analysis of soil samples

Soil pH and electrical conductivity (EC) were determined using a 1:2 soil-to-water ratio suspension and measured with a digital pH and conductivity meter, following the procedures outlined (31, 32). The organic carbon (OC) content in the soil was assessed using the wet digestion method developed by Walkley & Black (33). Available phosphorus (P) was estimated through Olsen’s method (34), while available nitrogen (N) was determined using the alkaline permanganate method proposed (35). The ammonium acetate extraction technique described (36) was employed to analyze available potassium (K). For micronutrient analysis including copper (Cu), iron (Fe), manganese (Mn), and zinc (Zn) the DTPA extraction method recommended (37).

### 2.4 Disease incidence and severity

Disease incidence and severity data were collected through direct field observations using standard visual rating scales. Observations were initially recorded using a categorical 0 to 9 scale, which quantifies the extent of visible symptoms on plant parts. These ordinal ratings were subsequently converted into percentage values to enable meaningful comparisons. To assess differences between NF and CF systems, an independent samples t-test was applied. This test was appropriate as the objective was to compare means between two independent groups (NF vs. CF). The analysis was conducted separately for each disease: Canker, Root Rot, Collar Rot, Alternaria, Marssonina, and Powdery Mildew across four distinct blocks: Chaupal, Jubbal, Nichar, and Pooh. The use of the t-test allowed us to statistically determine whether observed differences in disease levels between the two farming systems were significant.

### 2.5 Comparative economic analysis between NF and CF

The comparison of the cost of cultivation between NF and CF was conducted by considering all inputs/material costs, labor costs, and various types of variable costs. However, fixed cost concepts were excluded from the analysis. Focusing on variable costs allows for a direct comparison of the expenses directly related to production activities in natural farming versus conventional farming. This comparison can provide insights into the immediate cost differences between the two approaches.

### 2.6 Structural equation modeling approach

**Figure 2:**
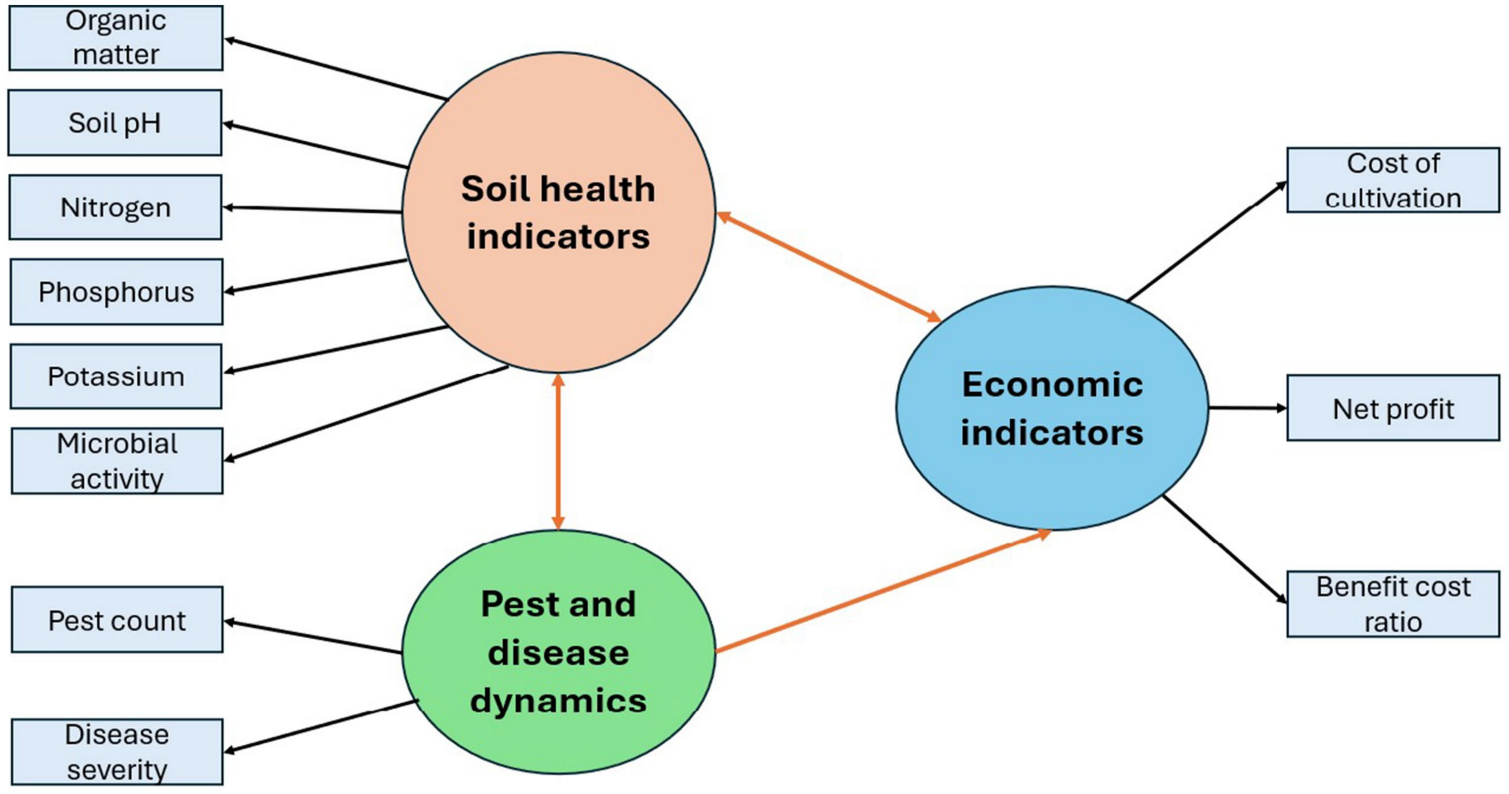
Conceptual framework.

Structural Equation Modeling (SEM) was utilized to examine the interrelationships among soil health indicators, pest and disease dynamics, and economic indicators (Figure 2). While SEM typically incorporates latent variables, this study focused on modeling the causal pathways exclusively among observed variables. This approach enabled the assessment of both direct and indirect effects among measurable indicators of soil health, pest and disease dynamics, and economic performance.

To investigate the interrelationships among soil health, pest and disease dynamics, and economic outcomes, a set of research hypotheses (H1 to H11) was developed based on theoretical insights and prior empirical evidence (Table 1). These hypotheses outlined the anticipated directions and nature of associations among the observed variables incorporated in the Structural Equation Modeling (SEM) framework. The SEM analysis, specifically using path analysis, was employed to test these hypotheses. Based on the results, hypotheses were accepted if the corresponding path relationships were statistically significant (*p* ≤ 0.05), and rejected if the relationships were not statistically significant (*p* > 0.05).

**Table 1:** Summary of observed variables used in SEM.

Variable	Measurement method	Expected sign	Set of hypothesis
Organic Matter	Laboratory Analysis	+	H1: Organic Matter positively influences microbial activity and soil health.
pH Level	Laboratory Analysis	±	H2: pH level has a non-linear effect on microbial activity and nutrient availability.
Nitrogen	Laboratory Analysis	±	H3: Nitrogen levels in the soil have a direct effect on disease severity and plant resilience.
Phosphorus	Laboratory Analysis	±	H4: Phosphorus positively influences plant growth and economic outcomes.
Potassium	Laboratory Analysis	±	H5: Potassium influences gross returns and the Benefit–Cost Ratio (B-C Ratio).
Microbial Activity	Laboratory/Field Assessment	+	H6: Microbial activity positively influences soil fertility and organic matter.
Disease Severity	Field Observation	−	H7: Disease severity negatively affects plant resilience and economic outcomes.
Cost of Cultivation	Farmer Interviews/Records	−	H8: The cost of cultivation negatively affects profitability (net profit and B-C ratio).
Gross Returns	Farm Records/Survey Data	+	H9: Gross returns positively influence net profit and the Benefit–Cost Ratio (B-C Ratio).
Net Profit	Derived from Returns & Costs	+	H10: Net profit positively affects the Benefit–Cost Ratio (B-C Ratio).
Benefit–Cost (B-C) Ratio	Computed from Data Collected	+	H11: Net profit positively affects the Benefit–Cost Ratio (B-C Ratio).

Soil Health (ξ₁) → Pest & Disease (η₁); Soil Health (ξ₁) → Economic Indicators (η₂); Pest & Disease (η₁) → Economic Indicators (η₂).

Thus, the structural equations are:

$$\eta ₁=\beta_{11}\;\;\xi_{1}+\delta_{1}$$

$$\eta ₂=\gamma_{21}\cdot \xi_{1+}\;\beta_{21}\cdot \eta ₁+\delta_{2}$$

β_11_, γ_21_, β_21_ are path coefficients from exogenous/other endogenous variables. δ1, δ2 are structural errors.

## 3 Results and discussion

### 3.1 Major crop combinations under natural farming and conventional farming systems

**Figure 3:**
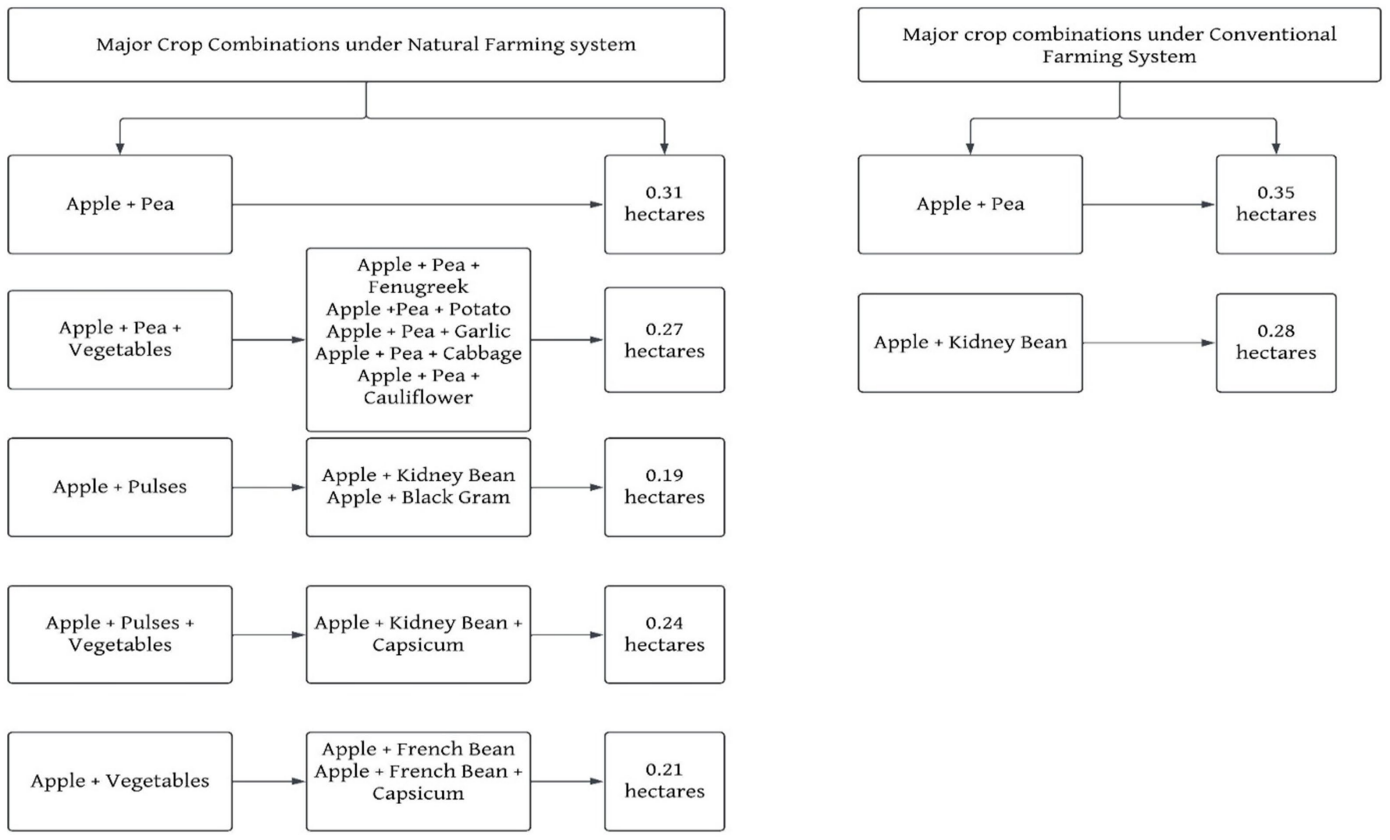
Major crop combinations.

Crop combinations significantly influence the productivity and sustainability of farming systems, particularly when comparing Natural Farming and Conventional Farming practices. In Natural Farming, the integration of diverse crop combinations, including legumes as intercrops, is strategically employed to optimize land utilization, enhance soil fertility, and boost yields through natural mechanisms such as nitrogen fixation (38, 39). Conversely, Conventional Farming predominantly adopts monocropping or limited crop rotations, relying heavily on external chemical inputs to maximize production. A comparative analysis of these systems underscores their distinct approaches to resource management, crop diversity, and environmental sustainability. The study revealed that farmers in the area cultivate various crops alongside apple plantations, employing diverse crop combinations under the Natural Farming system (Figure 3).

The primary combinations identified were: (i) Apple + Pea (average area: 0.31 hectares), (ii) Apple + Pea + Vegetables (0.27 hectares), (iii) Apple + Pulses (0.19 hectares), (iv) Apple + Pulses + Vegetables (0.24 hectares), and (v) Apple + Vegetables (0.21 hectares). These combinations reflect the farmers’ strategy to optimize land use through mixed cropping, which is vital for enhancing soil fertility and overall productivity.

In contrast, Figure 3 shows that farmers in the Conventional Farming system predominantly practiced monocropping, with only two crop combinations: (i) Apple + Pea (average area: 0.35 hectares) and (ii) Apple + Kidney Bean (0.28 hectares). Farmers often avoid intercropping in conventional apple plantations, focusing on maximizing apple yields. This approach relies heavily on chemical inputs for pest and weed management and favors simplified practices that enhance efficiency and reduce labor. Although this may increase short-term yields, it can lead to decreased soil health and biodiversity, jeopardizing the long-term sustainability of apple cultivation (40, 41).

### 3.2 Soil nutrient analysis

#### 3.2.1 Macronutrient analysis

The principle of NF posits that incorporating beneficial microbes can sustain crop yields without the use of synthetic fertilizers, as proponents believe the soil inherently contains all the essential nutrients required by plants (42–44). In this study, soil nutrient levels were compared between NF and CF systems to assess the impact of each method. To effectively compare the nutrient levels, box and whisker plots were employed. This method provides a comprehensive visual representation of the distribution of nutrient values, illustrating the median, interquartile range, and potential outliers for each nutrient analyzed.

**Figure 4:**
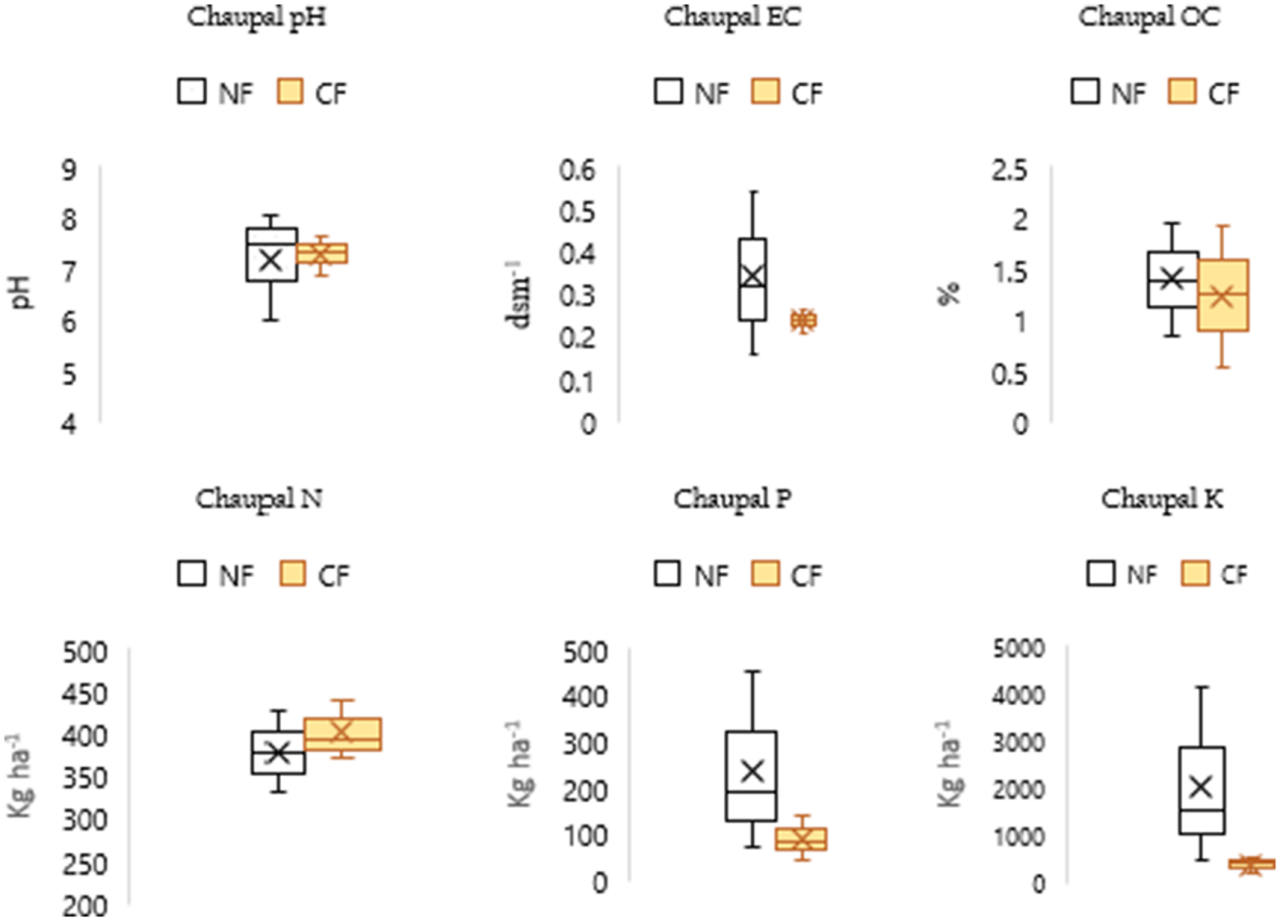
Comparative soil parameters under NF and CF systems in Chaupal block, showing higher OC content in NF soils (0.84–1.95%) compared to CF (0.53–1.91%), supporting NF’s positive role in improving soil fertility. Soil pH ranged from slightly acidic to slightly alkaline in both systems, while EC remained within safe limits for apple cultivation. NF also exhibited higher P and K availability despite lower N levels.

**Figure 5:**
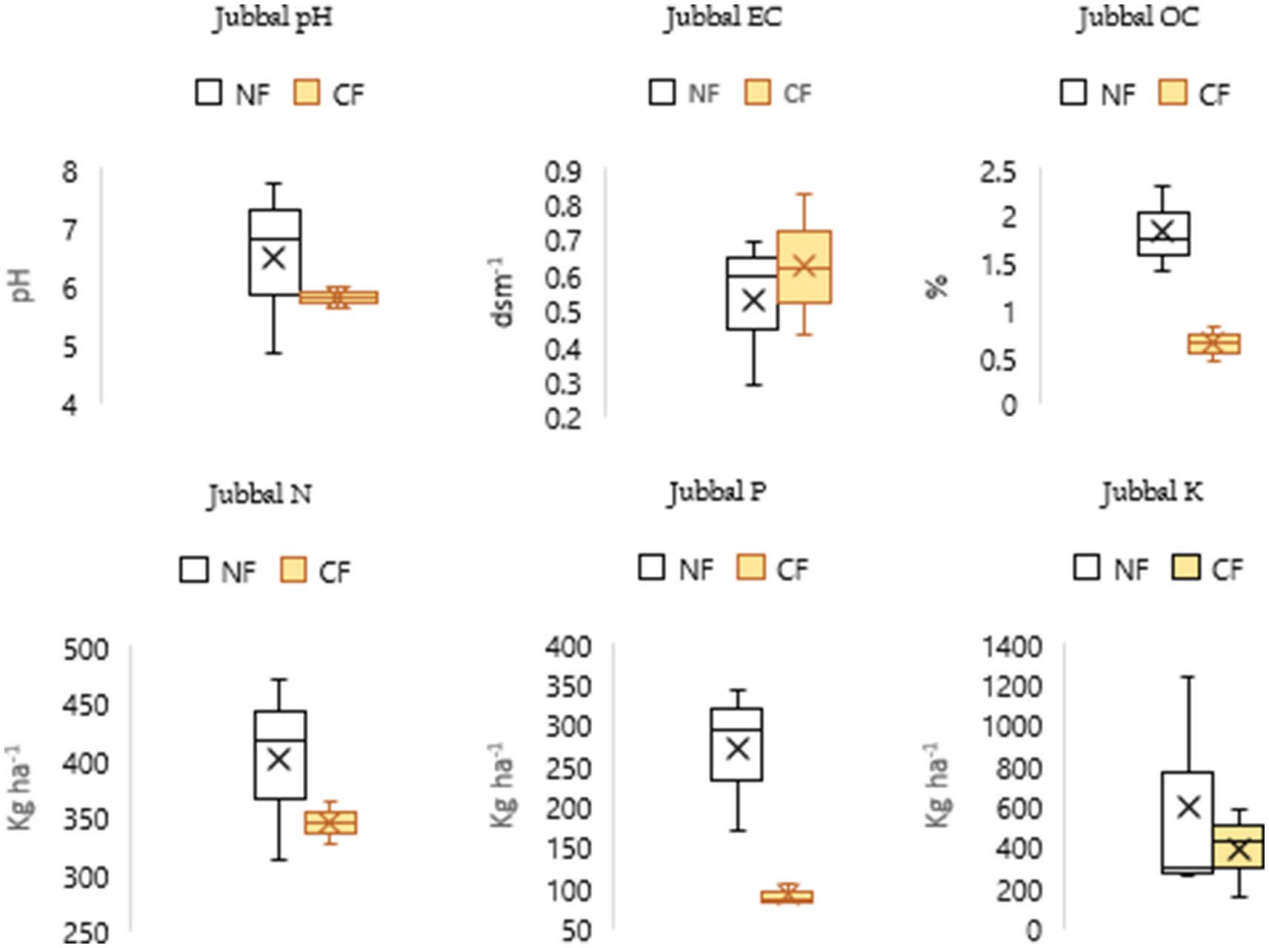
Soil quality indicators under NF and CF systems in Jubbal block, showing significantly higher OC (1.4–2.3%) in NF soils compared to CF (0.45–0.83%). Soil pH ranged more widely in NF (4.85–7.76), indicating higher variability, while EC remained within normal limits in both systems. Available N, P, and K were consistently higher under NF, reflecting its potential for enhancing soil fertility.

**Figure 6:**
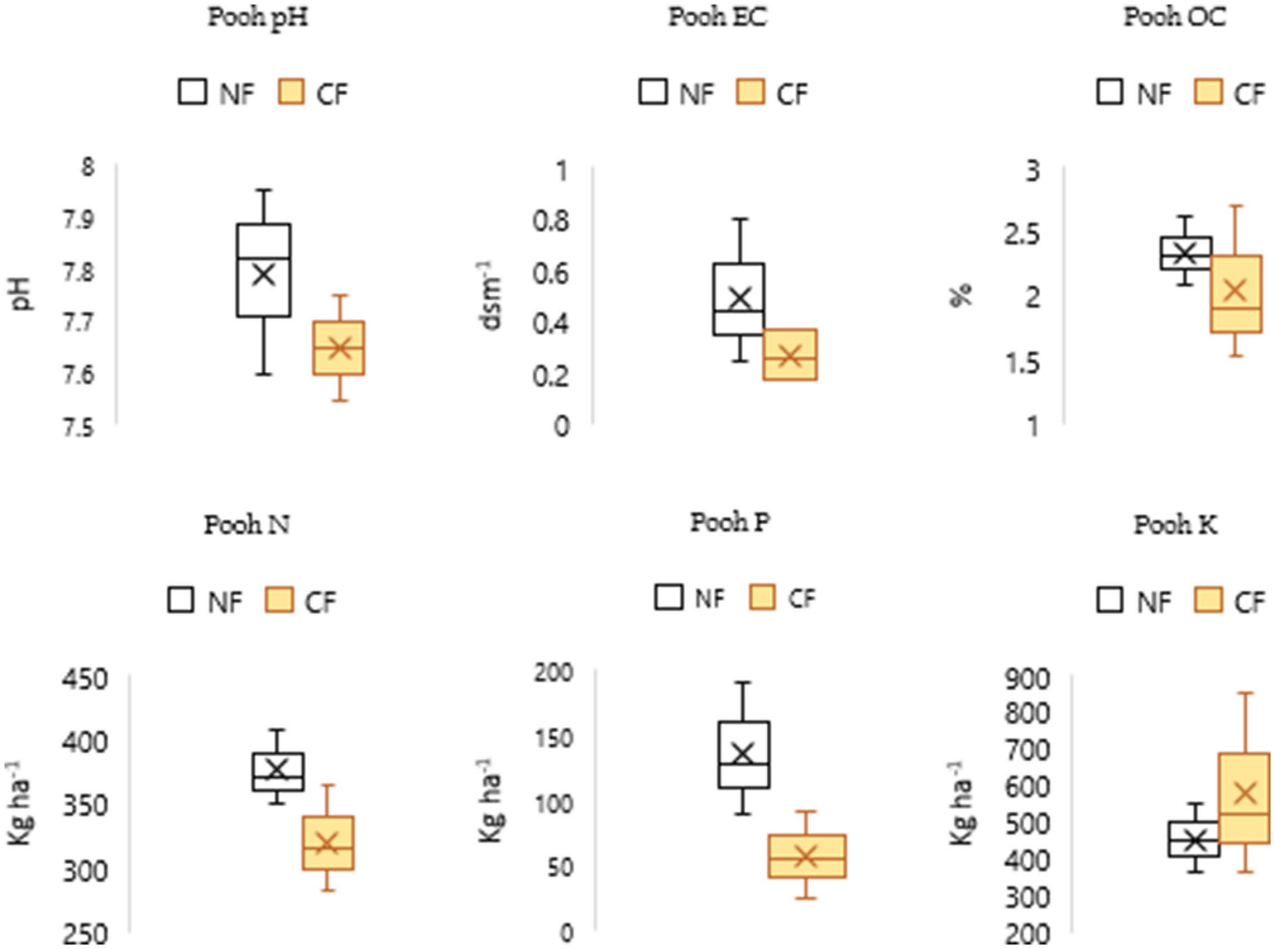
Soil parameters under NF and CF systems in the selected site, showing slightly alkaline pH and normal EC levels across both systems. OC content was very high in both NF (2.09–2.61%) and CF (1.53–2.7%) soils. Available N and P were higher under NF, whereas K levels were relatively lower, indicating differential nutrient dynamics between the two farming systems.

**Figure 7:**
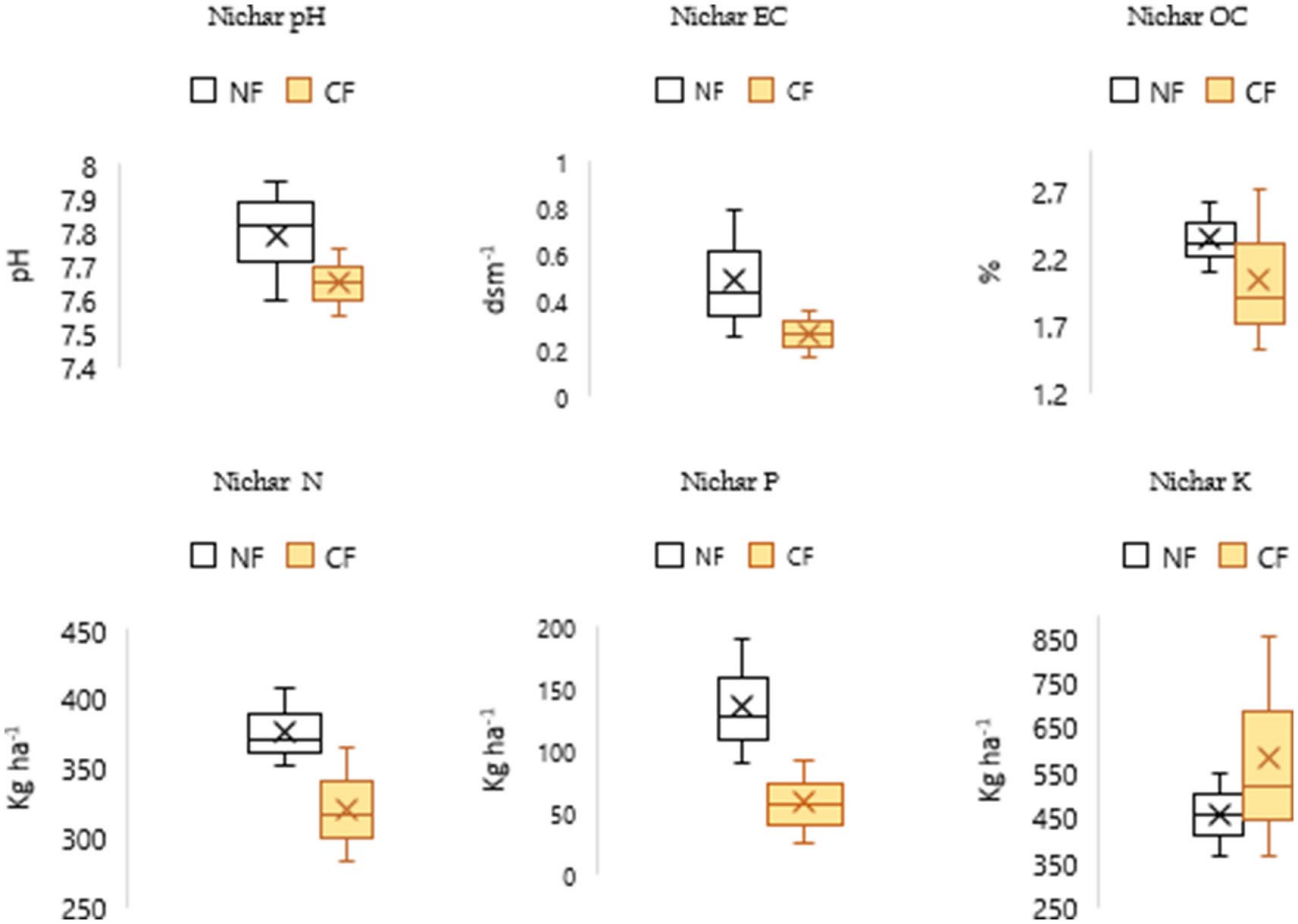
Soil characteristics under NF and CF systems in Nichar block, showing pH values ranging from medium acidic to neutral and EC levels within the normal range for both systems. OC content was high to very high in both NF (1.44–2.69%) and CF (1.32–2.34%) soils. Available N and P levels were higher under NF, while K was relatively lower.

The findings suggest that NF enhances organic carbon (OC) levels, contributing to improved soil fertility and long-term soil health across all blocks. Higher phosphorus (P) and potassium (K) levels under NF in the Chaupal (Figure 4) and Jubbal blocks (Figure 5), along with higher N and P levels in the Pooh (Figure 6) and Nichar blocks (Figure 7), indicate improved nutrient availability under NF, which may support better plant growth and yield. Notably, only the Jubbal block recorded higher levels of all three macronutrients N, P, and K under NF. In contrast, N availability was lower in the Chaupal block, while K was found to be lower in the Nichar and Pooh blocks. These observations highlight the need for targeted nutrient management, particularly for N and K, to optimize crop performance under NF while maintaining its sustainable and low-input principles.

To ensure the success of NF, there is a need for the adoption of a well-designed package of practices. This approach will enable optimal nutrient management, particularly for N and K, which were found to be lower in NF soils and are essential for plant growth. By effectively supplementing these nutrients while maintaining the core principles of sustainability and local resource utilization, NF can support enhanced crop health and productivity. These findings underscore the ecological sustainability of NF systems, demonstrating their capacity to maintain or even improve key agroecological indicators compared to CF systems. The improved soil health metrics under NF contribute to sustainable nutrient cycling, reduced external input dependence, and align with broader agroecological goals such as promoting ecological balance, resilience, and climate mitigation (45, 46).

#### 3.2.2 Micro-nutrient analysis

The micronutrient status of soils in Chaupal and Jubbal Blocks showed significant differences between Natural Farming (NF) and Conventional Farming (CF) systems (Table 2). In both blocks, NF had higher concentrations of key micronutrients such as zinc (Zn), copper (Cu), iron (Fe), and manganese (Mn) compared to CF. For instance, in the NF system, Zn ranged from 1.97 to 11.9 ppm, Cu from 0.91 to 27.8 ppm, Fe from 2.41 to 170 ppm, and Mn from 0.52 to 68.5 ppm. In contrast, the CF system displayed lower values, particularly for Zn and Mn.

**Table 2:** Micronutrient status of soil samples.

Chaupal block		NF (*n* = 10)	CF (*n* = 10)	Pooh block		NF (*n* = 10)	CF (*n* = 10)
Zn	Range	1.97–8.81	0.88–4.54	Zn	Range	3.83–7.83	5.81–8.63
	Mean ± SD	4.06 ± 2.73	2.71 ± 1.55		Mean ± SD	5.53 ± 1.91	7.26 ± 1.13
	SE	1.03	0.78		SE	0.95	0.51
Cu	Range	0.91–14.9	1.48–4.7	Cu	Range	1.19–11.5	1.33–3.59
	Mean ± SD	4.72 ± 4.97	2.62 ± 1.43		Mean ± SD	5.39 ± 4.35	2.62 ± 1.01
	SE	1.88	0.72		SE	2.18	0.45
Fe	Range	2.41–101	3.24–41	Fe	Range	7.21–12.2	8.93–15
	Mean ± SD	17.98 ± 36.70	13.52 ± 18.35		Mean ± SD	9.60 ± 2.30	11.61 ± 2.34
	SE	13.87	9.17		SE	1.15	1.05
Mn	Range	0.52–41.7	1.01–2.93	Mn	Range	1.69–3.54	1.96–14.8
	Mean ± SD	6.85 ± 15.38	1.66 ± 0.86		Mean ± SD	2.36 ± 0.82	6.02 ± 5.60
	SE	5.81	0.43		SE	0.41	2.5
Jubbal block		NF (*n* = 10)	CF (*n* = 10)	Nichar block		NF (*n* = 10)	CF (*n* = 10)
Zn	Range	5.45–11.9	2.19–4.17	Zn	Range	8.7–10.7	4.45–9.59
	Mean ± SD	8.36 ± 2.91	3.45 ± 1.78		Mean ± SD	9.67 ± 1.00	6.32 ± 2.83
	SE	1.45	1.26		SE	0.58	1.63
Cu	Range	1.73–27.8	2.36–4.18	Cu	Range	1.64–8.78	2.36–2.78
	Mean ± SD	9.1 ± 12.49	3.27 ± 1.29		Mean ± SD	4.50 ± 3.78	2.6 ± 0.21
	SE	6.24	0.91		SE	2.18	0.12
Fe	Range	10.7–170	32.6–91.7	Fe	Range	33.2–186	24–131
	Mean ± SD	55.52 ± 76.97	62.15 ± 41.79		Mean ± SD	86.93 ± 85.90	76.13 ± 53.55
	SE	38.48	29.55		SE	49.59	30.91
Mn	Range	1.46–68.5	8.64–41.1	Mn	Range	2.9–9.84	2.62–8.46
	Mean ± SD	19.04 ± 32.99	24.87 ± 22.95		Mean ± SD	5.41 ± 3.85	5.17 ± 2.99
	SE	16.49	16.23		SE	2.22	1.72

Micro-nutrient analysis from the soils of Pooh and Nichar Blocks showed higher levels of Zn, Cu, Fe, and Mn under Natural Farming (NF) compared to Conventional Farming (CF). NF soils in Nichar showed very high to extremely high levels of these nutrients, while CF soils had generally lower values. In Pooh, NF soils also recorded higher levels of Zn, Cu, and Fe compared to CF. The higher micronutrient availability in NF also improves crop resilience, leading to stronger growth, better disease resistance, and higher yields. Moreover, the long-term soil health benefits of NF contribute to sustained productivity over time. Organic farming systems, including Natural Farming, improved the availability of essential micronutrients such as zinc, iron, and manganese in the soil, which are critical for plant health and productivity (47).

### 3.3 Insect-pests and mite status

The pests considered in the study include Woolly Apple Aphid (*Eriosoma lanigerum*), San Jose Scale (*Quadraspidiotus perniciosus*), Apple Root Borer (*Dorysthenes hugelii*), Leaf Folder (*Archips termias*), and Mite (*Tetranychus* spp.; Table 3). In general, CF reported a lower incidence of Woolly Apple Aphid in blocks like Chaupal (20.92%), Nichar (17.5%), and Pooh (40.83%) compared to NF, where infestation levels were higher (30.43, 29.85, and 50.08%, respectively). On the other hand, the incidence of San Jose Scale was observed to be higher in NF than in CF across all blocks, except in Pooh, where CF exhibited a slightly higher value. This higher infestation under NF could be due to the absence of chemical pesticides, which typically control the scale insects (48). The incidence of Apple Root Borer (*Dorysthenes hugelii*) showed mixed trends across the blocks. In Chaupal, CF had slightly higher levels (34.33%) compared to NF (31.64%), whereas in Nichar, NF showed a significantly higher infestation (42.38%) compared to CF (32.38%). This variation may be attributed to NF’s reduced soil disturbance, which provides a more favorable environment for the root borer. Similarly, the infestation of Leaf Folder (*Archips termias*) was notably higher in Pooh under NF, with an incidence of 41% compared to just 5.5% in CF. Over time, however, balanced pest populations in NF systems are likely to emerge, aligning with the self-regulating ecosystem principles central to agroecological intensification (49).

**Table 3:** Incidence of major pests under NF and CF (%).

Blocks	Natural farming					Conventional farming				
	Woolly apple aphid	San Jose scale	Apple root borer	Leaf folder	Mite	Woolly apple aphid	San Jose scale	Apple root borer	Leaf folder	Mite
Chaupal	30.43	26.86	31.64	14.29	22	20.92	25.75	34.33	12.08	5.92
Jubbal	30.32	16.95	22.73	31.36	13	24.82	11.09	34	15.73	19.91
Nichar	29.85	20.54	42.38	13.69	26.15	17.5	10.25	32.38	6.63	22.88
Pooh	50.08	11.83	-	41	25.33	40.83	15	29.83	5.5	14

These findings indicate that while NF aligns with the ecological goals of agroecological intensification, it faces challenges in pest management due to the lack of synthetic interventions. However, this challenge can be addressed by integrating eco-friendly pest management practices within the NF framework. These practices may include the use of fermented plant extracts (e.g., Agniaster, Brahmaster, Neemaster), botanical biopesticides, crop diversification, and the enhancement of habitats for natural enemies. Strengthening farmer capacity in applying these natural solutions will be crucial to maintaining pest balance and ensuring the long-term sustainability of NF systems, without compromising their core principles of reducing external inputs and promoting ecological balance.

### 3.4 Disease incidence and disease severity

In terms of disease incidence, it was observed that NF generally results in lower disease occurrence compared to CF across all locations (Table 4). For canker, NF showed a lower incidence, particularly in Chaupal (10.58%) compared to CF (14.37%), and this trend was consistent across other locations. Similarly, for root rot, NF consistently results in lower incidence, especially in Jubbal, where CF shows a much higher rate of infection (8.52%) compared to NF (2.44%). Collar rot also follows a similar pattern, with NF showing significantly lower incidence in all locations compared to CF, especially in Chaupal, where the incidence is reduced from 1.87% in CF to 0.38% in NF. The statistical tests confirm these findings as statistically significant with *p*-values indicating a strong difference.

**Table 4:** Disease incidence of apple orchard (natural farming, chemical farming) in different districts of Himachal Pradesh (%).

Particulars			Chaupal	Jubbal	Nichar	Pooh
Disease Incidence	Canker	NF	10.58	7.57	3.45	6.19
		CF	14.37	9.07	2.55	5.33
		*t* value	4.08	2.26	2.29	2.36
		*p* value	0.0075	0.043	0.041	0.038
	Root rot	NF	3.24	2.44	3.86	3.62
		CF	5.5	8.52	1.78	4.16
		*t* value	5.95	15.02	10.92	2.51
		*p* value	0.002	0.000057	0.0002	0.033
	Collar rot	NF	0.38	2.21	0.6	1.6
		CF	1.87	3.57	0.06	3.5
		*t* value	13.79	3.77	2.33	3.59
		*p* value	0.00008	0.0097	0.039	0.011
Disease Severity	Alternaria	NF	24.95	11.8	8.84	8
		CF	22.82	18.11	4.19	1.83
		*t* value	2.97	10.82	8.69	10.53
		*p* value	0.02	0.0002	0.00048	0.00023
	Marsonina	NF	0.83	1.99	3.96	–
		CF	–	8.7	0.41	0.73
		*t* value	8.45	14.87	7.9	4.68
		*p* value	0.00053	0.000059	0.00069	0.0047
	Powdery Mildew	NF	–	1.53	–	–
		CF	0.25	0.5	0.33	–
		*t* value	7.77	3.48	4.47	–
		*p* value	0.00073	0.012	0.0055	–

For Alternaria, NF exhibits higher severity in Chaupal (24.95%), while CF shows increased severity in Jubbal (18.11%). In Nichar and Pooh, CF shows a significant increase in severity. For Marsonina, NF exhibits higher severity in Nichar (3.96%), but CF shows a higher severity in Jubbal (8.7%), with a notable lack of Marsonina cases in NF at Pooh, where CF has a mild presence (0.73%). Finally, Powdery Mildew was mostly absent in NF across locations, whereas CF showed some cases, particularly in Nichar (0.33%). The statistical analysis for these diseases supports significant differences between farming systems. Overall, the results suggest that Natural Farming tends to reduce both disease incidence and severity compared to Conventional Farming, with statistically significant differences observed in all locations (50–52).

### 3.5 Profitability analysis

Profitability analysis offers essential insights that enable farmers to make educated choices regarding the farming system that best aligns with their financial objectives and limitations. From the Table 5, it is evident that there is a 40.60% decrease in the total variable cost under NF (Rs. 106105.60) compared to CF (Rs. 178643.71). Several studies, including (53, 54) have documented a substantial reduction in the cost of cultivation for all crops under NF.

**Table 5:** Comparison of profitability in apple cultivation under NF and CF in Himachal Pradesh (Rupees in hectares).

Particulars	NF	CF	% change in NF over CF
Layout & Land Preparation	12524.76	19432.23	−35.55
	(13.17)	(10.88)	
Nutrition management (FYM, natural fertilizers/chemical fertilizers, micro nutrient sprays)	9376.44	45452.14	−79.37
	(9.86)	(25.44)	
Plant protection measures	13982.67	45872.30	−69.52
	(14.70)	(25.68)	
Labor	54599.00	45557.13	19.85
	(57.41)	(25.50)	
Miscellaneous expenses	4622.73	22329.91	−79.30
	(4.86)	(12.50)	
Total variable cost	95105.60	178643.71	−46.76
	(100)	(100)	
Yield (quintals/ha)	161.25	158.73	1.59
Average price per quintal	5,000	5,000	–
Gross income	806,250	793,650	1.59
Net returns	711144.40	615006.29	15.63

Figures in parentheses are percent of total variable cost.

Land preparation costs under NF were found to be 35.55 per cent lower (Rs. 12,524.76/ha) compared to CF (Rs. 19,432.23/ha). This reduction can be attributed to the presence of companion crops in NF apple orchards, which minimizes weed growth and consequently lowers expenses related to land management. The cost of nutrition management under NF amounted to Rs. 9,376.44/ha, representing a reduction of almost 80 per cent compared to CF, which incurred Rs. 45,452.14/ha. Similarly, plant protection expenses under NF totaled Rs. 13,982.67/ha, nearly 70 per cent lower than CF’s Rs. 45,872.30/ha. These significant cost savings were a major incentive for orchardists to transition to NF practices in their apple orchards. NF incurred higher labor costs (+19.85%) compared to CF, primarily due to its holistic and labor-intensive approach. Managing orchards without synthetic chemicals requires greater human intervention for tasks such as hand weeding, applying Jeevamrit and other natural formulations, and continuous pest monitoring and control. However, this increased labor expenditure was effectively offset by substantial input cost savings, particularly in nutrition and plant protection, which were nearly 80 and 70% lower, respectively, under NF than CF. Despite the modest yield gain of 1.59%, NF achieved higher gross income and a 15.63% increase in net returns over CF, driven largely by reduced cultivation costs. Furthermore, the reduction in external input dependency enhances system resilience, making NF more economically stable during market price fluctuations. Farmers practicing NF are less vulnerable to rising input costs or supply disruptions, thereby improving their financial security and adaptive capacity.

These findings therefore underscore that NF is not only environmentally sustainable but also economically viable, reinforcing its potential as a holistic agroecological approach. Several studies (15, 55–57) have similarly reported increases in both crop yield and net returns under NF, further validating its alignment with key agroecological principles such as economic sustainability, ecosystem health, and farmer livelihood enhancement.

### 3.6 Structural equation modeling

In this study, 11 hypotheses (H1 to H11) were initially developed to explore links among soil health, pest/disease dynamics, and economic outcomes using Structural Equation Modeling (SEM). However, only 6 paths were found statistically significant (*p* ≤ 0.05) and thus accepted (Table 6). The remaining hypotheses were found to be non-significant (*p* > 0.05) and excluded. As per standard SEM practice, only significant and meaningful paths are discussed.

**Table 6:** Estimated path coefficients from the SEM model.

S. No.	From → To	Path coefficient	Standard error	*z*-value	*p*-value	Hypothesis status
1.	Cost of cultivation → Net profit	−0.45	0.164	2.75	0.003	H8: accepted
2.	Net profit → Gross returns	0.93	0.283	3.29	0.001	H9: accepted
3.	Organic matter → Microbial activity	0.05	0.019	2.63	0.01	H6: accepted
4.	Nitrogen → Disease severity	−0.05	0.025	−1.99	0.047	H3: accepted
5.	Potassium → Gross returns	−0.45	0.18	−2.5	0.012	H5: accepted
6.	Potassium → B-C ratio	−0.62	0.234	−2.65	0.008	H5: accepted

**Figure 8:**
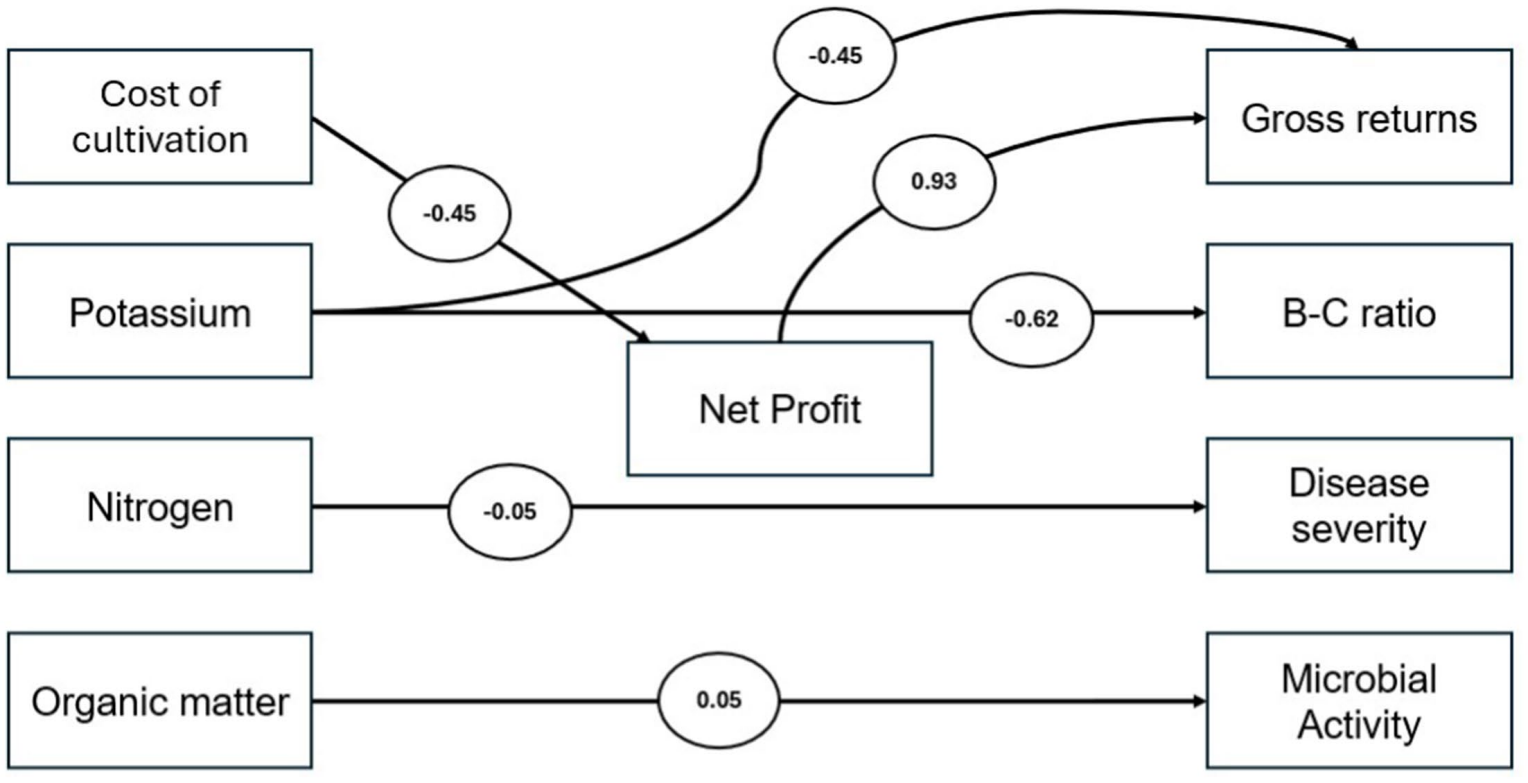
Path diagram of Structural Equation Modeling (SEM).

The SEM analysis revealed six statistically significant paths (p ≤ 0.05) among the variables examined (Figure 8). The cost of cultivation negatively impacted net profit (*β* = −0.45, *p* = 0.003), indicating that rising input costs reduce profitability. In contrast, net profit had a strong positive influence on gross returns (*β* = 0.93, *p* = 0.001), suggesting that improving profitability directly enhances revenue from apple cultivation. These results underscore the need for a well-designed package of practices under Natural Farming (NF) in the region to optimize resource allocation and manage input costs effectively. Organic matter significantly predicted microbial activity (*β* = 0.05, *p* = 0.01), indirectly supporting soil fertility and profitability, thereby reinforcing the ecological-economic synergy of NF and underscoring the importance of biologically rich soils in promoting beneficial microbial communities (58, 59). Nitrogen levels were found to reduce disease severity (*β* = −0.05, *p* = 0.047), highlighting the role of balanced nitrogen management in improving plant health and resilience (60, 61). However, excessive potassium application negatively affected both gross returns (*β* = −0.45, *p* = 0.012) and the Benefit–Cost (B-C) ratio (*β* = −0.62, *p* = 0.008), suggesting economic inefficiencies associated with overuse (62). Overall, the findings highlight the critical role of balanced nutrient management and call for the development of a region-specific NF package of practices that enhances soil health, controls pest and disease incidence, and improves economic outcomes in apple cultivation.

## 4 Policy implications

The study provides important policy insights to facilitate the widespread adoption of NF practices in apple cultivation. To effectively support this transition, a structured policy roadmap is recommended:

### 4.1 Strengthening capacity-building initiatives for NF practices

Government should prioritize the development of comprehensive training programs that equip farmers with practical knowledge on the preparation and use of natural inputs such as *Jeevamrit*, *Beejamrit*, and organic mulching materials. These programs must also cover nutrient management techniques and eco-friendly pest control strategies, with content tailored to regional agro-ecological conditions. Enhancing agricultural extension services can ensure timely delivery of this knowledge at the grassroots level.

### 4.2 Financial support through credit and incentive mechanisms

To ease the financial burden associated with adopting and sustaining NF, targeted policy support should include subsidies, low-interest loans, and tailored insurance schemes for NF practitioners. Since many farmers already practice intercropping with legumes, vegetables, and maintain crop diversity, these efforts should be strengthened through continued financial incentives and technical assistance. Support for input preparation should focus on facilitating access to essential raw materials like cow dung, cow urine, and locally sourced herbs. This can include subsidies for constructing compost pits, fermentation units, storage tanks, and other on-farm infrastructure required to prepare natural formulations. Encouraging community-based input preparation centers or shared resource units could also enhance accessibility and reduce individual costs.

### 4.3 Developing market linkages for NF apple produce

Ensuring stable and profitable market access for NF apples is essential to support long-term adoption. Strengthening institutional frameworks like the existing NF- CETARA (Natural Farming Certified Evaluation Tool for Agriculture Resource Analysis) certification system, which is already operational in the state, should be a priority. Efforts should focus on expanding the coverage of this system, streamlining certification processes, and raising farmer awareness about its benefits. Additionally, building robust market linkages through the formation of Farmer Producer Organizations (FPOs), establishment of local aggregation and distribution hubs, and support for branding and labeling of NF certified produce can enhance visibility and consumer trust. Facilitating access to premium markets such as organic retail chains, government procurement programs, and digital platforms can significantly improve economic returns for NF apple growers.

## 5 Conclusion

The comparative study of NF and CF revealed clear differences in agronomic practices, ecological outcomes, and economic viability. NF promoted diversified cropping systems and improved soil health through increased organic matter content and balanced nutrient availability. Although a slightly higher pest incidence was observed during the initial transition from chemical-based to ecological pest regulation, NF systems exhibited lower disease severity and greater system resilience. Economically, NF reduced input costs and increased net returns, demonstrating its potential as a sustainable alternative for apple cultivation. Structural Equation Modeling (SEM) identified organic matter and microbial activity as key contributors to profitability. It also revealed that imbalances in nutrient levels, particularly elevated potassium concentrations, were associated with reduced economic efficiency, suggesting a need for improved nutrient monitoring even under natural input regimes. These findings support the development of region-specific NF practices that optimize ecological processes and enhance long-term soil and economic health. Future research should explore the long-term effects of NF across different altitudinal zones and assess its scalability across diverse fruit-based farming systems.

## Data Availability

The raw data supporting the conclusions of this article will be made available by the authors, without undue reservation.
